# Genetic diversity and differentiation among *Prosopis alba* (Leguminosae) populations from dry valleys of Bolivia with different levels of human disturbance and altitude

**DOI:** 10.1002/ece3.4610

**Published:** 2018-10-31

**Authors:** Cecilia Bessega, Carolina Pometti, Ramiro Pablo López, Daniel Larrea‐Alcázar, Reneé H. Fortunato, Beatriz Saidman, Juan Cesar Vilardi

**Affiliations:** ^1^ Departamento Ecología, Genética y Evolución, Facultad de Ciencias Exactas y Naturales Universidad de Buenos Aires Buenos Aires Argentina; ^2^ Instituto de Ecología, Genética y Evolución (IEGEBA) CONICET‐Universidad de Buenos Aires Buenos Aires Argentina; ^3^ Facultad de Ciencias Puras y Naturales, Carrera de Biología Universidad Mayor de San Andrés La Paz Bolivia; ^4^ Asociación Boliviana para la Investigación y Conservación de Ecosistemas Andino‐Amazónicos (ACEAA) La Paz Bolivia; ^5^ N. Repetto y Los Reseros s.n., Hurlingham (1686) Argentina ‐ Consejo Nacional de Investigaciones Científicas y Técnicas (CONICET) Instituto de Recursos Biológicos (CIRN–CNIA, INTA) Buenos Aires Argentina

**Keywords:** altitude, bottleneck, genetic diversity, homozygote excess, SSR

## Abstract

The fast expansion of human population around La Paz, Bolivia (3,200–4,100 m.a.s.l.) triggered new suburban settlements in nearby areas in valleys and mountain feet. The white mesquite, *Prosopis alba* Griseb. (Leguminosae), is a resource (originally used by native communities) that is strongly affected by changes in land use. A gradient in the level of disturbance is found moving away from the La Paz city toward less altitude areas. The main objective of this study was to characterize genetically three *P. alba* populations with different levels of human disturbance located at different altitudes in Bolivia, in order to provide some guidelines for management and conservation of these species. Based on 10 SSR loci, the populations showed high level of genetic diversity in comparison with other forest species. The population less disturbed and situated at the lowest altitude was the most variable (*H*_e_ **= 0.51–0.42), whereas the less variable was the most disturbed and situated at the highest altitude. Heterozygote excess was observed in all populations. Most of genetic diversity (99%) is contained within populations. Genetic differentiation among populations is low (1%), suggesting low gene flow among populations. No evidence of recent bottlenecks events was detected. The estimates of the effective population size were low in all populations. The results are in agreement with the hypothesis that genetic diversity is reduced by the impact of anthropic disturbance in the population located at higher altitude in comparison with the lightly disturbed situated at lower altitude and farther from urban settlements.

## 1 INTRODUCTION

Highland landscapes are constituted by heterogeneous topography and environmental gradients that characterize the habitats (Körner, 2003). Steep valleys and mountain ridges demarcate the plant population habitats where gene flow is reduced favoring an important differentiation among populations (Hafdıs Hanna, Kuss, & Stöcklin, 2009). Genetic differentiation can be also increased by drift associated with reduced population size and founder effects. In consequence, population parameters in mountain regions may highly differ from those characterizing non‐isolated areas occurring at lower altitudes. About 35% of the Bolivian territory is covered by the tropical Andes. Biodiversity resources available above height of 1,000 m m.a.s.l. has been influenced and transformed in several ways by historic processes. The native composition of the high Andes population in Bolivia includes, among others, Aymara, Quechua, Uru, and Kallawaya nations, although significant admixture has occurred among these communities. The biodiversity found in the agricultural areas constitutes the basis of food safety and sovereignty of the Andean populations. From pre‐hispanic colonization, they had developed important crop species such as the quinoa *Chenopodium quinoa *and the potato *Solanum tuberosum*, but many other plant species have been domesticated for different uses, such as medicines, construction, fuel and spiritual activities (Vidaurre, Paniagua, & Moraes, 2006). Moreover, Bolivian communities sited in valleys and mountain foots in Tarija, Chuquisaca and La Paz, use wood from native forest species such as the “aliso” (alder tree, *Alnus acuminata*), “algarrobo blanco” (white mesquite, *Prosopis alba*) and “tipa colorada” (*Pterogyne nitens) *to make punts, ax handles, and utensils like dishes and spoons (Beck, Paniagua, & Yevara, 2001; McKean & Robinson, 1996; Nagashiro, 1992; Villanueva, 1997).

One of the most important cities in Bolivia is La Paz, which is situated between 3,200 and 4,100 m.a.s.l. The human population that is fast growing triggered new suburban settlements around the city and nearby areas in valleys and mountain feet, affecting biodiversity and the resources originally used by native communities. The white mesquite that grows in the dry valleys of Bolivia is one of the species most affected by this change in land use. It is possible to find a gradient in the level of disturbance of the tree populations when someone moves away from La Paz city toward less altitude areas.

The effects of urbanization on forest populations are comparable to those produced by overexploitation and land conversion into crop plantations in terms of habitat fragmentation, mating system changes, and gene flow restriction (Cascante, Quesada, Lobo, & Fuchs, 2002; Jump & Penuelas, 2006). When populations become genetically isolated, they are at risk of losing the genetic diversity that is critical to their long‐term survival (Sork & Smouse, 2006). As pointed before, this effect can be exacerbated if it is considered that the populations that are growing in the valleys might be expected to show some degree of isolation as they are geographically isolated and gene flow may be reduced because dispersals may not be able to move such enough to maintain the populations in contact. As a consequence of the isolation, it is expected an immediate loss of alleles due to the reduction of the population, with the consequence of inbreeding, population divergence increase, and genetic diversity reduction within population patches (Lowe, Boshier, Ward, Bacles, & Navarro, 2005; Young, Boyle, & Brown, 1996). However, the longevity of the trees and effective seed and pollen dispersal (when possible) can enhance their resistance to the negative effect of the forest fragmentation (Hamrick, 2004; Jump & Penuelas, 2006).

The degree of environmental disturbance is rapidly increasing in the neighborhoods of La Paz in Bolivia, but its effects on the genetic structure of *P. alba* and other important dry‐valley plant species populations are still unknown. Taking into account, the importance of this resource in arid and semiarid regions assessing its genetic structure is paramount to provide some guidelines for management and conservation. The main objective of this study was to examine the level of genetic diversity of populations of *P. alba* with different levels of human disturbance located in valleys at different altitudes in Bolivia. We characterized the population genetic structure, evaluated the possible occurrence of recent bottlenecks events, and estimated the effective population size in each sampling site. Through these analyses, we addressed the following questions: (a) Is there difference in the genetic diversity level found in the populations at different altitude? (b) Are genetic diversity parameters affected by the level of disturbance? (c) Are populations located in different valleys significantly differentiated from each other? (d) Have the population undergone recent bottleneck processes? v) What are the population effective sizes? We compared the results obtained in these populations with previous reports on two *P. alba* Argentinean populations (Campo Duran and Fernandez‐Forres) (Bessega, Pometti, Ewens, Saidman, & Vilardi, 2016) situated at much lower altitudes (150–450 m.a.s.l) with different levels of disturbance produced by agricultural activities.

The study hypothesis is that Bolivian *P. alba* populations situated at higher altitudes and highly disturbed areas will have lower genetic diversity within populations than those inhabiting less disturbed and lower altitude regions. Furthermore, gene flow restrictions among different valleys would determine significant genetic differentiation among population patches. The results of this study might contribute to decision making in *P. alba* management and conservation programmes in the dry valleys of Bolivia.

## 2 MATERIAL AND METHODS

### 2.1 Study species and genotyping

*Prosopis alba* is a very important tree from an ecological and economical point of view in arid and semiarid regions of South America and particularly in Argentina and Bolivia. It is widely distributed but forests are being intensely damaged. There are two important causes that affect the studied populations, first local communities and local people use this resource because of its important properties, the wood exhibits high quality (Pometti et al., 2009), it is used for carpentry and floors, and the fruits are considered a good resource as forage and for human use (Roig, 1993). Second, human activities are damaging the remaining forest. On the one hand, the agricultural frontier is growing and on the other, human populations are growing and expanding and native areas are being damaged while populations become fragmented. Moreover some populations are placed in valleys and genetic isolation may become important worsening the effect of human perturbation on trees populations.

**Figure 1:**
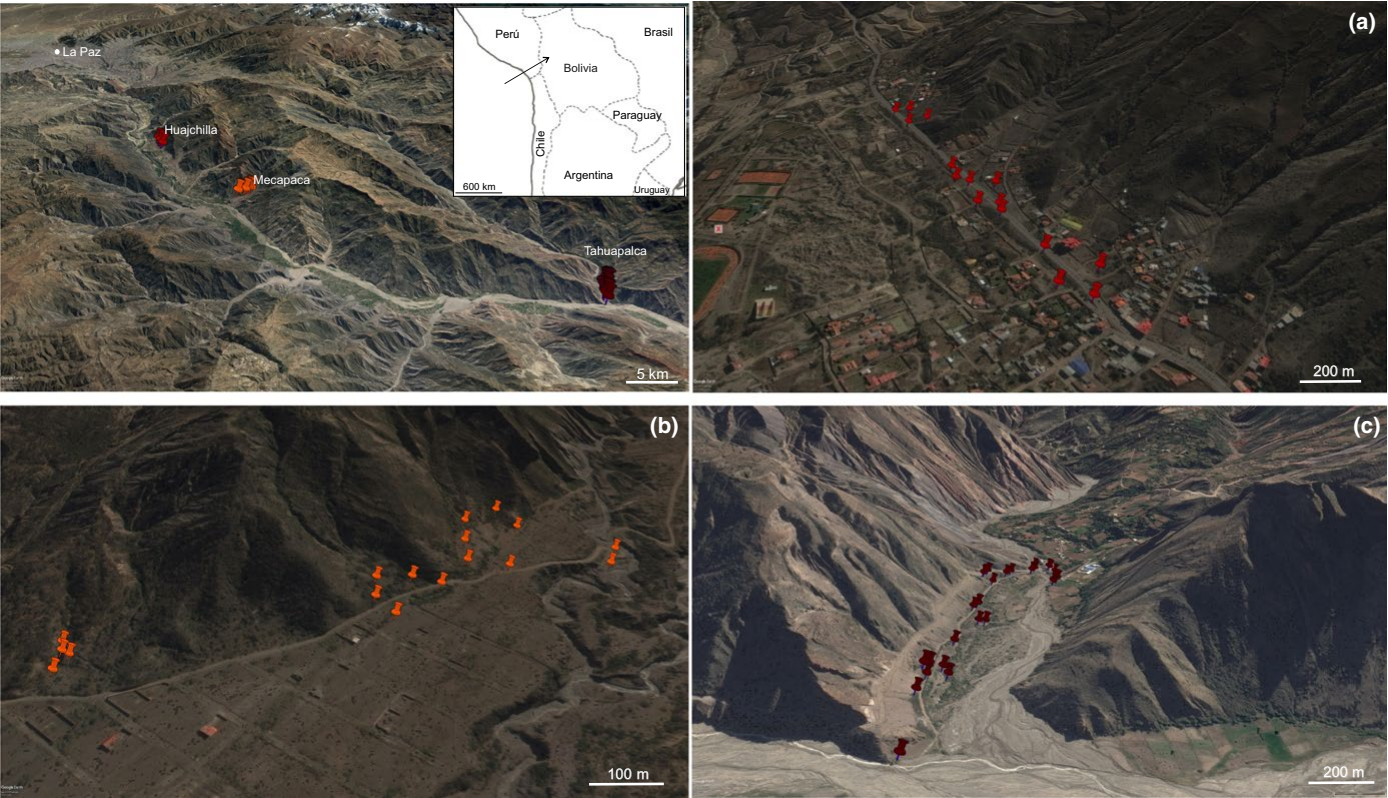
Geographical location of the sampled populations of *Prosopis alba *in Bolivia and enlarged maps showing spatial distribution in Huajchilla (a), Mecapaca (b), and Tahuapalca (c). Note: Images are from Google earth Pro (https://www.google.es/earth/)

We collected fresh young leaves samples from three populations of *P. alba* located at different altitude in the dry valleys of La Paz Bolivia (Figure 1). Voucher specimens were identified according to the proposal of Burkart (1976) and each sampled tree are deposited at the INTA Castelar BAB herbarium, Buenos Aires, Argentina (BAB N° 93,019 to 93,080). These populations differ from each other in the intensity of human disturbance. The most affected is Huajchilla (3,051 m.a.s.l.), located about 23 km *SSE* of La Paz city in a very populated area. Mecapaca (2,900 m.a.s.l.), ~10 km *SE* of the former has a lower human population density and disturbance, with evidence of incipient urban projects. Tahuapalca (2,300 m.a.s.l.), about 18 km *SE* of Mecapaca, is accessed following a hard‐winding road and shows almost no evidence of anthropic activities and environmental disturbance.

Collection method followed Bessega et al. (2000), Bessega, Pometti, Ewens, Saidman, and Vilardi (2011). Based on pollen and seed dispersal estimates in *P. alba* Argentinean populations, these authors recommend sampling trees separated more than 50 m from each other in order to avoid collecting genetically related material. From 15 to 30 adult individuals adult trees were sampled in each location. The two populations closest to La Paz were relatively small and sampling size corresponded to the highest possible following the criteria applied. Population size in Tahuapalca was grater but trees were located on the slope of the mountain making collection very difficult. A total of 61 adult individuals were studied from the three populations.

Total genomic DNA was extracted using the Qiagen DNeasy Plant kit (Qiagen, Valencia, CA) from leaf material that was collected and stored in silica gel. The samples were genotyped at 10 microsatellite markers described by Mottura, Finkeldey, Verga, and Gailing (2005), Mo05, Mo13, Mo08, Mo09, and Bessega et al. (2013), GL8, GL18, GL24, GL15 GL12, and GL6. The PCR amplifications were carried out in a 50 μL reaction volume containing 10–30 ng DNA, 0.6 μM each primer, 0.2 mM dNTPs, 0.3 U Taq DNA polymerase (Invitrogen, Buenos Aires, Argentina), and 1.5 mM MgCl 2. A Applied Biosystems Veriti™ Thermal Cycler was used for amplifications, where the cycling profile was initial denaturation at 94°C for 5 min; followed by 30 cycles at 94°C for 45 s denaturation, primer‐specific annealing temperature (58–62°C) for 45 s, and at 72°C for 45 s extension; and a final extension at 72°C for 10 min. PCR products were electrophoresed by Macrogen company (KOREA) and automatically sized using GENEMARKER version 1.91 (SoftGenetics LLCTM www.softGenetics.com).

### 2.2 Genetic diversity estimates

Linkage disequilibrium was tested by the index of association (*Ia*) and a slightly modified statistic which is independent of the number of loci (*rd*) (Agapow & Burt, 2001). These coefficients are based on the comparison between the observed variances of the “distance” between all pairs of individuals with the expected under linkage equilibrium. Both coefficients were estimated with the package *poppr* (Kamvar, Brooks, & Grunwald, 2015; Kamvar, Tabima, & Grunwald, 2014) of R program (R Core Team, 2017). Significance of *Ia* and *rd* was estimated by shuffling genotypes at each locus by a permutation test with 1,000 replicates.

Genetic diversity was quantified in each population through the number of alleles (*N_a_*), allelic richness (*A_r_*), the number of private alleles (*P_a_*), observed heterozygosity (*H*_o_) and expected (*H*_e_) and unbiased Nei's gene diversity (u*H*_e_) (Nei, 1978) using the package diveRsity (Keenan, McGinnity, Cross, Crozier, & Prodohl, 2013) for R software (R Core Team, 2017) using the *rarefaction* option as the sample size of the populations was not equal. Homozygote excess was quantified by the fixation index (*F*_IS_). Diversity measures and *F*_IS_ were compared among populations and considering nonoverlapping 95% confidence intervals as significance criterion. The possibility that heterozygotes deficiency may be due to null alleles was tested by Monte Carlo with 1,000 randomizations as described by Guo and Thompson (1992) and the U statistic described by Rousset and Raymond (1995) with 10,000 using the software ML‐Null (Kalinowski & Taper, 2006).

### 2.3 Population structure and differentiation

Genetic differentiation among populations was estimated by *G*_ST_ (Nei & Chesser, 1983) and *G*_'STH _(Hedrick, 2005) using Genalex 6.5 (Peakall & Smouse, 2006; 2012). Nei and Chesser *G*_ST_ is widely applied, shows a straightforward relationship to gene flow and mutation rate, and is useful for comparison with other studies. However, as *G*_ST_ may underestimate diversity in multiallelic loci, diversity was also evaluated by *G*_'STH_ which is most appropriate for SSR. The 95% confidence intervals were calculated using 1,000 bootstraps as implemented in Genalex 6.5. Total diversity (*H*_T_) was partitioned into among populations (*G*_ST_ × *H_T_*) and within population (*H_S_*) using Nei and Chesser (1983) approach. The estimates of genetic differentiation were compared with those from Argentinean reanalyzing the original matrix of Bessega et al., (2016) using only the same loci amplified in the Bolivian populations and randomly sampled individuals to get similar size matrices.

To identify population structure in Bolivian populations a Bayesian model‐based cluster analysis was performed using the STRUCTURE program version 2.3.4 (Pritchard, Wen, & Falush, 2009). We explored which value of K maximized the likelihood of the data. The burn‐in period and the number of Monte Carlo Markov chain (MCMC) repetitions were set, respectively, to 25,000 and 50,000 and *K* values were averaged across ten iterations. Considering the range of populations and distribution both admixture and no‐admixture models with correlated allele frequencies were used. *K* was set from 2 to 4, and the optimum *K* value was identified as the run with the highest likelihood value, following the recommendations of Pritchard, Stephens, and Donnelly (2000). We also performed the same analysis including the two Argentinean populations from Bessega et al. (2016), Fernandez (FF, Sgo del Estero) y Campo Duran (CD, Salta), with different level of perturbation setting K from 2 to 6.

Finally, differentiation among populations was evaluated by two methods. The first one was Discriminant Analysis of Principal Components (DAPC), using the *adegenet *package (Jombart, 2008; Jombart & Ahmed, 2011) (function *dapc*) of software R (R Development Core Team, 2017). This analysis was performed with prior information on individual populations. The second method was a Canonical discriminant analysis to SSR data to summarize variation between predefined classes (populations) for classification variables (alleles presence/absence). This analysis was carried out with the software Statistica 5.5 (StatSoft Inc., 2012).

### 2.4 Population bottleneck and effective population size

To detect evidence of recent bottleneck in each population, we used BOTTLENECK 1.2.02 software (Piry, Luikart, & Cornuet, 1999). The software computes for each population sample and for each locus the distribution of the gene diversity expected from the observed number of alleles (*k*) given the sample size (*n*) under the assumption of mutation‐drift equilibrium. To determine whether populations exhibit a significant number of loci with gene diversity excess we used a sign test, the standardized difference (Cornuet & Luikart, 1996) and the Wilcoxon rank test (Luikart, Allendorf, Cornuet, & Sherwin, 1997). We assumed the two phases mutation model (TPM) as this model is recommended for microsatellites markers (Di Rienzo et al., 1994). This model considers mostly one‐step mutations but also a small percentage of multistep changes (Luikart & Cornuet, 1998).

We assessed the effective population size in populations of *P. alba* using the linkage disequilibrium method (LDNe) of Hill (1981) as implemented in NeEstimator V2.1 (Do et al., 2014; Waples & Do, 2008) that includes the Waples (2006) bias correction. For *Ne*_LD_, we used the criterion *P*_crit_ = 0.02 which gives a good balance among accuracy and bias (Waples & Do, 2008). Confidence Intervals for Ne_LD_ were based in the scaled *X* ^2^ distribution implemented in the software. *Ne* was also calculated from the population average coancestry (*θ*) as *Ne_θ_* = 0.5/*θ* (Cockerham, 1969); being *θ* the average coancestry coefficient between all pairs of individuals using the J. Nason estimator (Loiselle, Sork, Nason, & Graham, 1995) estimated using SPAGEDI (Hardy & Vekemans, 2002). Finally, *Ne* was also estimated by the relation proposed by Long (1986) *Ne*_F_ = −0.5*F*_IS_.

## 3 RESULTS

### 3.1 Genetic diversity

The 10 loci analyzed were variable with 2–9 alleles in Tahuapalca population and 1–5 alleles in Mecapaca and Huajchilla (Table 1). The privates alleles detected were much higher in Tahuapalca (*P_a_*=16) than in the remaining populations (*P_a_* = 2). The allelic richness (*A_r_*) also differs significantly between Tahuapalca and the remaining populations as the 95% confidence intervals do not overlap. Mean *A_r_*in Tahuapalca was 3.9 [CI_95%_ = 3.1–4.2], 2.99 [CI_95%_ = 2.6–3] in Mecapaca, and 2.99 [CI_95%_ = 2.4–3] in Huajchilla. The mean expected heterozygosity (*H*_e_) was similar in Mecapaca and Huajchilla (*H*_e_ = 0.44 and 0.42) but higher in Tahuapalca (*H*_e_ = 0.51).

**Table 1:** Sample size (*N*), no. alleles (*N_a_*), allelic richness (*A_r_*), private alleles (*P_a_*), observed heterozygosity (*H*_o_), expected (*H*_e_) and unbiased expected heterozygosity (*uH*_e_), inbreeding coefficient (*F*_IS_), *p*(null): probability of heterozygote deficiency due to null alleles tested by Monte Carlo (1,000 replicates)

Pop	Locus	*N*	*N* *_a_*	*A* *_r_*	*P* *_a_*	*H*_o_	*H*_e_	u*H*_e_	*F*_IS_	*p*(null)
Tahuapalca	Mo05	30	2	2	0.00	0.47	0.42	0.43	−0.111	–
	Mo13	30	5	4.25	2.00	0.63	0.63	0.64	−0.012	–
	Mo08	30	2	2	0.00	0.87	0.50	0.51	−0.741	–
	Mo09	30	5	4.73	1.00	0.53	0.66	0.67	0.189	0.017
	GL8	29	5	3.45	4.00	0.17	0.19	0.20	0.105	–
	GL18	30	3	2.99	0.00	0.43	0.45	0.45	0.026	–
	GL24	30	5	4.64	2.00	0.47	0.48	0.49	0.028	–
	GL15	30	9	6.75	4.00	0.3	0.69	0.70	0.564	0.000
	GL12	30	8	6.60	3.00	0.73	0.73	0.74	−0.004	–
	GL6	29	3	2.48	0.00	0.48	0.41	0.42	−0.182	–
Mean		29.8	4.	3.99	1.60	0.51	0.51	0.52	−0.014	–
*SE*		0.13	0.75	0.75	0.52	0.06	0.05	0.05	0.103	–
Mecapaca	Mo05	16	2	2	0.00	0.5	0.38	0.39	−0.333	–
	Mo13	16	3	3	0.00	0.56	0.63	0.65	0.103	–
	Mo08	15	2	2	0.00	0.47	0.36	0.37	−0.304	–
	Mo09	15	5	5	1.00	0.93	0.67	0.7	−0.386	–
	GL8	16	1	1	0.00	0	0	0	–	–
	GL18	14	2	2	0.00	0.07	0.07	0.07	−0.037	–
	GL24	16	3	3	0.00	0.31	0.53	0.54	0.405	0.004
	GL15	16	5	5	0.00	0.5	0.71	0.73	0.295	0.008
	GL12	16	3	3	0.00	0.75	0.55	0.57	−0.357	–
	GL6	16	4	3.99	1.00	0.31	0.49	0.51	0.363	0.008
Mean		15.6	3	3	0.20	0.44	0.44	0.45	−0.028	–
*SE*		0.22	0.42	0.42	0.13	0.09	0.08	0.08	0.104	–
Huajchilla	Mo05	15	2	2	0.00	0.93	0.5	0.51	−0.875	–
	Mo13	15	3	3	0.00	0.8	0.65	0.67	−0.229	–
	Mo08	15	3	3	1.00	0.6	0.44	0.45	−0.371	–
	Mo09	15	4	4	1.00	0.87	0.64	0.66	−0.349	–
	GL8	15	1	1	0.00	0	0	0	–	–
	GL18	15	3	2.93	0.00	0.2	0.18	0.19	−0.084	–
	GL24	15	2	2	0.00	0.2	0.18	0.19	−0.111	–
	GL15	15	4	4	0.00	0.47	0.66	0.68	0.288	0.019
	GL12	15	5	5	0.00	0.8	0.66	0.69	−0.208	–
	GL6	14	3	3	0.00	0.29	0.3	0.31	0.059	–
Mean		14.9	3	2.99	0.20	0.52	0.42	0.44	−0.209	–
*SE*		0.1	0.37	0.37	0.13	0.1	0.08	0.08	0.102	–
Weighted mean		–	–	–	–	–	–	–	−0.066	–

Observed heterozygosity (*H*_o_) was similar to *H*_e_ in Mecapaca (*H*_o_ = 0.44) and Tahuapalca (*H*_o_ = 0.51) whereas in Huajchilla *H*_o_ (0.52) was higher than *H*_e_. Average *F*_IS_ estimates were negative in all populations as well as their weighted mean (Table 1) suggesting heterozygote excess. However, the result is significant only in Huajchilla where the CI does not contain the zero. Despite this trend to heterozygote excess, some loci showed heterozygote deficiency in some populations. In these cases, the presence of null alleles was tested with the software ML‐Null. The results were significant only for the locus GL15 which might have null alleles in the three populations. The exclusion of this locus does not produce significant changes in global *F*_IS_ estimates.

The analysis of linkage disequilibrium in the whole sample yielded significant results (*p* = 0.04), which are attributable to Tahuapalca only (*p* = 0.026), as in the other two populations the association index was non‐significant. However, after applying Bonferroni correction for multiple tests linkage disequilibrium was not significant in any case.

### 3.2 Population structure and differentiation

The population structure results showed that the highest proportion of genetic diversity is contained within individuals (99%) and the remaining variation by the among population component (1%). The global estimations of genetic differentiation were *G*_ST_ = 0.029 and *G*'_STH_ = 0.068 (Table 2 first column). The 95% CI does not contain the zero indicating significant population differentiation among the Bolivian populations. The level of genetic differentiation was similar to that obtained for Argentinian populations (Table 2 second column). However, the among population differentiation considering the Bolivian and Argentinian populations is near seven times higher than the level detected within a region (*G*_ST_ = 0.209 and *G*'_STH_ = 0.523) (Table 2 last column) indicating a significant differentiation among populations from different countries.

**Table 2:** Global estimates of genetic differentiation

	Among Bolivian pops	Among Argentinean pops^a^	Among All
*G*_ST_	0.029 [0.011–0.049]	0.018 [0.008–0.026]	0.209 [0.133–0.281]
*G*'_S__TH_	0.068 [0.021–0.123]	0.087 [0.035–0.158]	0.523 [0.363–0.626]

Note

*G*_ST_ (Nei & Chesser, 1983) and G'_S__TH_ (Hedrick, 2005).

a Values among Argentinean populations are based on a random same size matrix from data belonging to Bessega et al. (2016) based on the same 10 SSR loci. [CI_95%_].

**Figure 2:**
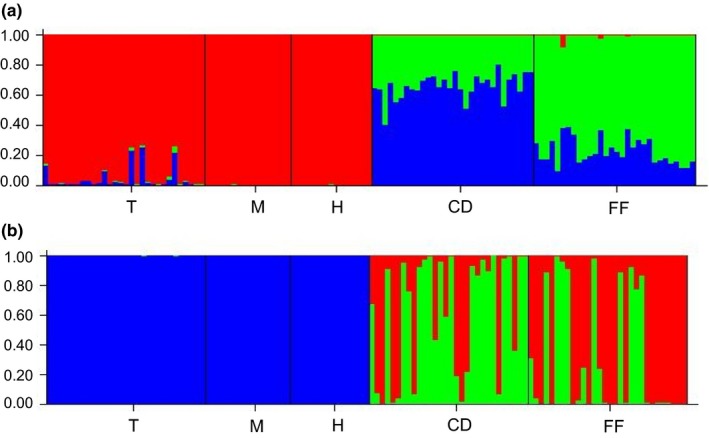
Clustering of individuals made by STRUCTURE for *K* = 3 considering admixture (a) and no‐admixture (b) models. Each individual is represented by a vertical bar that is partitioned into colored segments that represent the individual's estimated membership fractions. Same color in different individuals indicates that they belong to the same cluster. Note: T: Tahuapalca (Bolivia); M: Mecapaca (Bolivia); H: Huajchilla (Bolivia); CD: Campo Duran (Argentina); FF: Fernandez (Argentina)

Bayesian analyses performed with STRUCTURE did not show population structure for Tahuapalca, Mecapaca, and Huajchilla populations considering either the admixture or the no‐admixture models (*K* = 1, ln *P*(*X*/*K*) = −1,160.00 and −1,160.02, respectively). The lack of evidence of genetic discontinuities suggests no influence of historical events for these populations. When the analysis is performed adding the Argentinean populations, the number of clusters identified was *K* = 3 (ln *P*(*X*/*K*) = −3,098.00 and −3,087.06 for admixture and no‐admixture models). One of these clusters is represented by the trees collected in the three Bolivian populations, Tahuapalca, Huajchilla, and Mecapaca (Figure 2). The remaining two clusters comprise Campo Duran and Fernandez previously studied Argentinean populations respectively (Bessega et al., 2016), independently of the model used.

**Figure 3:**
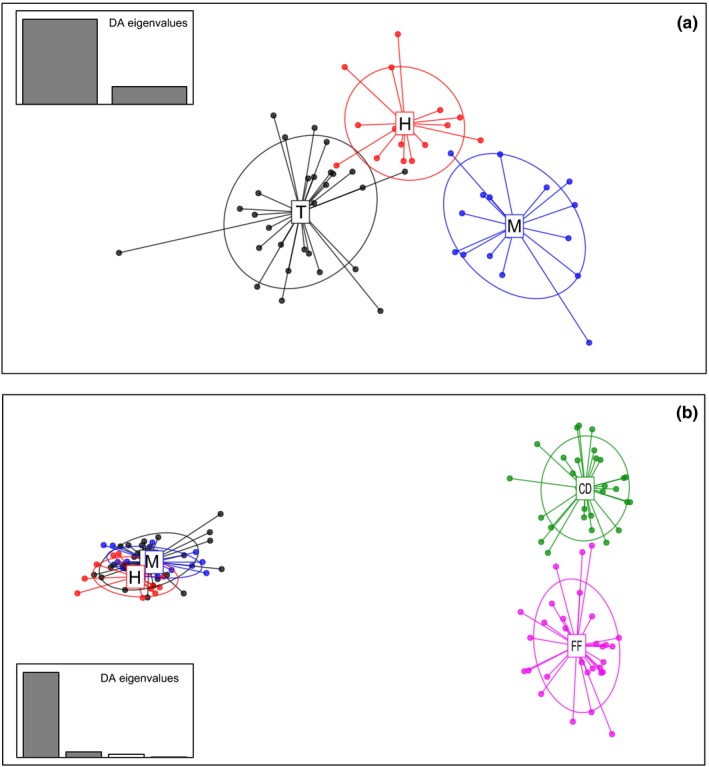
Scatterplot of individuals on the two principal components of DAPC. (a) Bolivian populations (b) Argentinean and Bolivian populations. The graph represents the individuals as dots and the groups as inertia ellipses. Note: T: Tahuapalca (Bolivia); *M: *Mecapaca (Bolivia); H: Huajchilla (Bolivia); CD: Campo Duran (Argentina), FF: Fernandez (Argentina)

**Figure 4:**
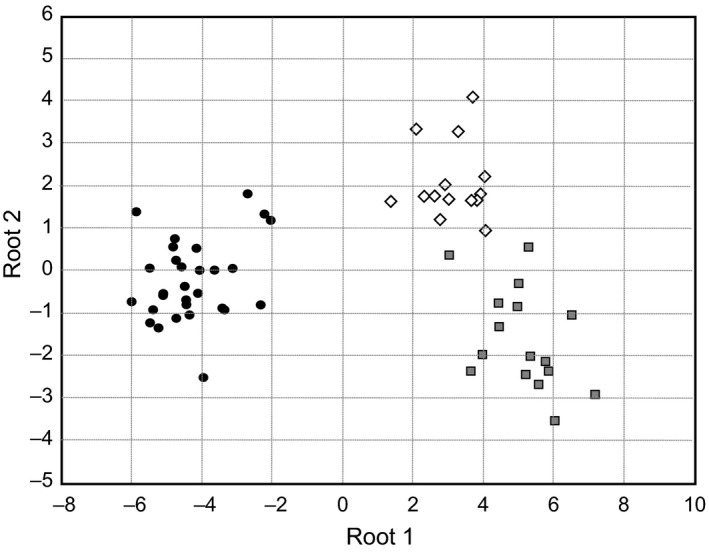
Plot of canonical discriminant functions 1 and 2 of *P. alba* populations, Huajchilla (white diamond), Mecapaca (gray square), and Tahuapalca (black circle) from SSR data

In the DAPC analysis, two axes were retained explaining 100% of total variation (respectively 82.79% and 17.21%) (Figure 3a). The scatterplot shows that the three a populations are clearly differentiated and only a few individuals from Tahuapalca overlaps with those from Huajchilla (Figure 3a). The first PC differentiates Tahuapalca from the other two populations (*p* < 2 × 10^−16^), whereas Huajchilla is differentiated from Mecapaca by the second PC (*p* = 1.15 × 10^−7^). The correspondence between the prior and the posterior classification of individuals in their respective populations varied from 93% in Huajchilla to 96% in Tahuapalca. The Canonical discriminant analysis based on the presence and absence of alleles was consistent with those of the DAPC (Figure 4). The first and second canonical axes accounted for 87.5% and 12.5% of the variation, respectively, explaining all the genetic diversity. A high correspondence was also observed between prior and posterior classification averaging 93%. Nine SSR alleles were sufficient to differentiate populations (*p* < 0.05). When DAPC is performed adding the Argentinean population, the tendency observed by global differentiation estimators (Table 2) is clearly visualized (Figure 3b). The two first axes comprise about 96% of total variation (90% and 6% for PC1 and PC2 respectively). The differentiation between populations from Argentina and Bolivia is much higher than the differentiation found among Bolivian populations. The differentiation found between the two previously studied populations of Argentina is higher than that among Bolivian populations in concordance with Table 2.

### 3.3 Population bottleneck and effective population size

The results of the analysis of recent bottleneck were not significant for the three populations according to any of the tests used, indicating that the loci analyzed do not depart from neutrality and the populations have not undergone a recent bottleneck (Table 3). The results of estimation of effective population size by LDNe (*Ne*_LD_) showed low values in Tahuapalca (*Ne*_LD_ = 38) and Mecapaca (*Ne*_LD_ = 88), but failed to yield an estimate in Huajchilla based on the SSR analyzed loci (Table 3). The estimation of *Ne* based on group coancestry (*Ne_θ_*) was only possible in Huajchilla where *θ* resulted positive. The effective size estimation in this population is small, *Ne_θ_* = 72.9 (Table 3). *N_e_* estimated from *F*_IS_ (*Ne*_F_) was 35.71, 17.85, and 2.39 for Tahuapalca, Mecapaca, and Huajchilla, respectively.

**Table 3:** Statistical tests of genetic bottlenecks under the two‐phase model (TPM), and effective population size (Ne) estimates obtained by the linkage disequilibrium (Hill, 1981) and multilocus coancestry coefficient (*θ*) (Cockerham, 1969) methods in *Prosopis alba* from the dry Valley of Bolivia

	Population		
	Tahuapalca	Mecapaca	Huajchilla
Mean *N*	59.6	31.2	29.8
Mean *k*	4.7	3	3
Mean* H*_e_	0.52	0.45	0.44
Sign test *p*‐value	0.524	0.154	0.600
Standarized differences test *p*‐value^a^	0.149	0.157	0.365
Wilcoxon Sign rank deficiency test *p*‐value^b^			
1 tail	0.577	0.180	0.367
2 tail	0.922	0.359	0.754
*Ne*_LD_ c	37.7	87.9	∞
CI [95%]	19.6–126.2	10.5 ‐∞	15.9–∞
*θ* d	−0.0005	−0.0015	0.0068
*Ne* *_θ_* e	–	–	72.9

Notes

Mean *N* = mean number of alleles, Mean *k*: mean number of alleles per locus.

a Cornuet and Luikart (1996).

b Luikart et al. (1997).

c *Ne*_LD_ estimated by NeEstimator V2.1 (Do et al., 2014).

d Coancestry within population (*θ*) estimated by Loiselle et al. (1995).

e *Ne* *_θ_* estimated as 05/*θ* (Cockerham, 1969).

## 4 DISCUSSION

Disturbance of natural habitats may lead to a decrease in population size and changes in the genetic structure of populations (Lowe et al., 2005). Anthropic activities including urban or peri‐urban settlements in formerly wild areas and the intensive use of woodlands may lead to a significant reduction and disturbance of natural populations. This is the case of *P. alba* forests which are overexploited for wood and nonwood (edible pods) resources. As original local communities are still dependent on the native resources, they are strongly affected by the expansion of urban boundaries and the change of human habits. Forest fragmentation is expected to yield geographic isolation and decline of genetic diversity within populations associated with genetic drift or inbreeding (Grivet, Sork, Davis, & FW, 2008; Kramer, Ison, Ashley, & Howe, 2008). The “algarrobo blanco” woodlands in the dry valleys near La Paz (Bolivia) nowadays occur as patchy remnants embedded in a matrix of urban/suburban land. La Paz city is situated at high altitude (3,200–4,100 m.a.s.l.) and when moving away from the city, both the level of disturbance and altitude decrease.

For biological communities occurring in mountainous areas, altitude changes represent a series of physical factors that affect the establishment and survival of different populations. Indeed, geographic barriers, rainfall, and temperature may influence genetic diversity and population structure (Manel et al., 2012; Mosca et al., 2012; Shen, Bo, Xu, & Wu, 2014). The populations of *P. alba* situated in Bolivian highlands showed relatively high levels of genetic diversity, comparable to others woody trees species with the same life form (Hamrick, 1996; Hamrick, Godt, & Sherman‐Broyles, 1992; Nybom, 2004) but lower values than other *P. alba* populations growing at lower altitudes in Argentina (mean *H*_e_ = 0.45, *A_r_* = 3.3 and *H*_e_ = 0.67, *A_r_* = 11.03, respectively) (Bessega et al., 2016).

A remarkable difference among the populations situated near La Paz was found. Tahuapalca, the less disturbed population situated at 2,300 m.a.s.l., turned out to be the most variable with the highest heterozygosity and number of private alleles, but it exhibited the lowest heterozygote excess. By contrast, Huajchilla situated at 3,050 m.a.s.l, the most disturbed population, shows the lowest allelic richness, number of private alleles and heterozygosity, and the highest heterozygote excess. Different causes might be claimed for this result. A plausible explanation is related to differences in effective population sizes. As a general trend, Bolivian valleys separated by mountains represent semi‐isolated population patches with relatively small effective sizes (*Ne* < 100) (as estimated with all applied methods). LDNe method is considered very robust (Wang, 2016) but can lead to downwards bias of *Ne*_LD_ when sample size (*N*) is small relative to *Ne* (England, Cornuet, Berthier, Tallman, & Luikart, 2006). The alternative approaches to estimate *Ne* are based on average pairwise coancestries (Cockerham, 1969) or heterozygosity excess (Long, 1986). The latter also yielded small effective sizes for all three populations. The coancestry estimates by Loiselle et al. (1995) may downward biased because negative values were obtained, which did not allow to apply Cockerham (1969) method in Tahuapalca and Mecapaca. In Huajchilla, where *θ* estimate was positive, the Ne estimate was also relatively low in consistence with the other approaches. The reduced population sizes are compatible with the location of these populations in semi‐isolated valleys and with the smaller populations in more disturbed locations. The heterozygote excess detected in *P. alba* from Bolivian highlands was similar to that detected in *Picea mexicana *(*F*_IS_ **= −0.107) (Ledig, Hodgskiss, & Jacob‐Cervantes, 2002) and *Podocarpus parlatorei* (*F*_IS_ = −0.104) (Quiroga & Premoli, 2007) which inhabit high elevation environments and compatible with the fact that Huajchilla population is the more disturbed one that exhibits the lowest effective size compatible with human disruption.

Allelic richness is a parameter that tends to respond rapidly to habitat loss or fragmentation and the remaining parameters are expected to have a higher temporal lag in the variation (da Silva Carvaho, 2015; Keyhonbadi, Roland, Matter, & Stobeck, 2005). Habitat perturbation can take longer to affect heterozygosity (Collevatti, Grattapaglia, & Hay, 2001), and it is also possible that population size of our studied populations has not yet affected severely this index (Kramer et al., 2008). These trees have very long generation times and it is possible to think that the effect of the reduction of the genetic diversity parameters is also being obscured by this phenomenon. It can be also considered that habitat loss and fragmentation in this region is a relatively recent process and it is possible that not all the variability estimates had become affected yet. Indeed, the variability estimates from Tahuapalca (2,300 m.a.s.l.) are similar to those populations from Argentina previously studied. Two *P. alba* Argentinean populations (Fernandez‐Forres and Campo Duran) studied by Bessega et al. (2016) did not exhibit differences in genetic diversity parameters in spite of the fact that they differ in the level of anthropic disturbance.

In recent bottleneck, the rare alleles are the first to be lost diminishing the mean number of alleles per locus. In contrast, heterozygosity is less affected generating a transient excess of heterozygosity compared to that expected given the resulting numbers of alleles (Cornuet & Luikart, 1996; Luikart & Cornuet, 1998; Maya Garcia et al., 2017). A reduction in genetic diversity is predicted during forest movement as a result of bottlenecks occurring throughout the range expansion (Newton, Allnut, Gillies, Lowe, & Ennos, 1999; Williams & Arnold, 2001). Both components make the hypothesis of a northwards expansion toward highlands for *P. alba*, plausible. Here, although we found a reduction in the genetic diversity parameters in the population at the highest altitude, we failed to found bottlenecks evidence in the populations studied. This may be explained by the long generation interval of *P. alba*. According to Cornuet and Luikart (1996), the maximum heterozygosity excess associated with the bottleneck effect occurs at about 0.5 × (2*Ne*) generations after the population size reduction (considering loci with 3–5 alleles). That means that in Tahuapalca the number of generations required to detect the maximum heterozygosity excess would be between 35–38 generations (based on *Ne* estimates obtained for this population from *F*_IS_ and with NeEstimator). As *P. alba* trees of more than 300 years old have been recorded, it is not unexpected that possible bottlenecks produced by human disturbance are too early to be detected.

The decrease in the genetic diversity, seen as lower number of alleles, can also be interpreted as an adaptation to altitude. This finding is not novel, as there are examples in the literature where variation in trees is reduced with altitude, as in *Nothofagus pumilio* populations (Premoli, 2003) and *Cryptomeria japonica* (Taira, Tsumura, Tomaru, & Ohba, 1997). However, other trees like *Populus szechuanica var tibetica* (Shen et al., 2014), *Euptelea pleiospermum* (Wei, Meng, & Jiang, 2013) and *Quercus aquifolioides* (Zhang, Korpelainen, & Li, 2006) do not exhibit differences in variation level along altitudinal gradients.

Most of the genetic variance in *P. alba* populations from Bolivia is occurring within populations (99%). The percent of genetic variance associated with among population differentiation, although significant, was low (1%). The genetic differentiation among populations was significant and both DAPC and canonical discriminant analysis clearly differentiate the individuals from different populations. On the other hand, the STRUCTURE analysis showed no structure for these populations and admixture within populations was only observed in Tahuapalca. The analysis with the admixture model shows some genetic similarity of Tahuapalca with the Argentinean populations studied by Bessega et al. (2016).

The relatively low genetic differentiation among Bolivian populations is compatible with the occurrence of some gene flow among valleys and/or short divergence times (in terms of number of generations). In *P. alba* seeds are dispersed endozoically by mammals (Campos et al., 2012; Mares, Enders, Kingsolver, Neff, & Simpson, 1977; Reynolds, 1954), and pollen is dispersed by insects (Genisse et al., 1990), both conditions usually associated with limited dispersal. Some species as the Andean fox (*Lycalopex culpaeus*) in the Valley of La Paz are able to disperse seeds in dungs (Maldonado, Pacheco, & Saavedra, 2014) at medium distances although the effectiveness for the dispersal services for *P. alba* had been discussed (Maldonado, Loayza, Garcia, & Pacheco, 2018). Big mammals such as the guanaco (*Lama guanicoe*) were described as possible disperser of *P. flexuosa* and *P. chilensis* (Campos et al., 2012), species highly related to *P. alba*. According to Bolgeri (2016), the guanacos in Mendoza (Argentina) are able to perform seasonal altitudinal migratory movements using during winter lower areas than in summer. Although camelids seem not to be present at these altitudes in Bolivian valleys, introduced mammals as horses, donkeys, and cows might be able to disperse *P. alba* connecting different valleys in Bolivia.

Relative low differentiation due to short population's histories has been claimed in *Picea glauca*, where insufficient time for evolutionary forces to differentiate populations was proposed (Alden & Loopstra, 1987). Also, in *Embothirum coccineum*, Mathiasen, Rovere, and Premoli (2007) found that genetic variation was not correlated with population and assumed that fragmentation events had occurred in relatively recent times, and the equilibrium between drift and mutation has not been reestablished. In the present case, the analysis of genetic differentiation adding the Argentinean populations together with those situated in Bolivia showed that the level of global differentiation among countries is near seven times higher. The Bolivian populations are much more homogeneous, and the only one showing some genetic similarity with the Argentinean populations is Tahuapalca. Populations at higher elevations tend to be genetically impoverished (Premoli, 2003) probably as a consequence of the combined effects of genetic drift and/or increased inbreeding rate. The possible effects of altitude and disturbance on genetic structure of populations in the Bolivian valleys cannot be discriminated because in the present study these factors are not independent. However, a similar level of genetic diversity was observed between two Argentinean populations with different disturbance level (Bessega et al., 2016). Probably the disturbance has not yet produced detectable effects on genetic diversity due to the long generation time of *P. alba* in comparison with the time elapsed from the start of human disturbance. For this reason, it is possible to assume the differences detected among populations in the Bolivian highlands are mainly determined by adaptation to altitude and/or historical events related with range expansion of *P. alba* from a southern original distribution. Two centers of diversity have been described for the genus *Prosopis*, the Mexican‐Texan and Chaqueño (Burkart, 1976). According to the presented results, it is possible to assume a radiation northward of *P. alba* from the Chaco diversity center in Argentina to lower latitudes and higher altitudes. This is compatible with the range‐expansion hypothesis that predicts (Williams & Arnold, 2001) the lower level of genetic diversity detected in the Bolivian populations.

To better understand the relationship between Argentinean and Bolivian populations, however, we should conduct a future genetic analysis on populations from central and southern Bolivia, which should show a lot more in common with Argentinean populations due to the fact that these populations are potentially in contact along a wide corridor of dry ecosystems that unites both countries.

The identification of management units (MU) from genetic data was reviewed by Palsbøll, Berube, and Allendorf (2006), and the delimitation of MUs upon the amount of genetic divergence demonstrated to be valuable for conservation purposes. Following Hastings (1993), a valuable criterion to assign populations to different MUs is a rate of dispersal among populations lower than 10%. Based on the genetic differentiation gene flow estimate would range between 3.4 and 8.3 migrants per generation (respectively based on *G*_STH_ and *G*_ST_) among Bolivian populations. As these values are higher than 10% of *Ne* estimates, they may be considered as belonging to a single management unit. By contrast, gene flow between Bolivian and Argentinean populations would be less than one migrant per generation suggesting that each area should be treated as different MUs.

## 5 CONCLUSIONS

The information obtained here has important implications for conservation and management purposes. Tahuapalca, the population situated at lower altitude and the minimal disturbance by anthropic activities has the higher diversity, what makes it worth protecting. Recently, a minimum effective population size (*Ne*) of 70 has been suggested to avoid inbreeding depression for in situ conservation (Caballero, Bravo, & Wang, 2016). Based on this, it is recommended to preserve also the populations with higher effective size here detected. On the basis of topology of the landscape and isolation due to valley occupation, the populations would probably tend to be much more differentiated than in plain landscapes. This condition makes also ex situ conservation highly recommended. As most of the genetic diversity is found to be contained within individuals, the collection should include many fruits from each tree to increase the contribution of more pollen donors as stated for *P. alba* in Bessega et al. (2011). As *Prosopis* forest historically provide resources used by native communities, protection policies should be recommended applying monitoring strategies to ensure the rationale use of *P. alba* avoiding non‐sustainable harvesting for immediate economic benefits or habitat destruction for construction purposes which might jeopardize the ecosystems as a whole.

## CONFLICT OF INTEREST

The authors declare that they have no conflict of interest.

## AUTHOR CONTRIBUTIONS

Conception or design of the work: C. Bessega, B. Saidman, J.C. Vilardi. Formal analysis and Methodology: C. Bessega, C. Pometti, J.C. Vilardi. Funding acquisition: C. Bessega, B. Saidman, J.C. Vilardi. Investigation: C. Bessega. Resources: C. Bessega, C. Pometti, R. Fortunato, RP Lopez, DM Larrea Alcazar. Interpretation of data for the work: C. Bessega, C. Pometti, R.P. López, D.M. Larrea‐Alcázar, R. Fortunato, B.O. Saidman, J.C. Vilardi. Writing—original draft: C. Bessega, C. Pometti, R.P. López, D.M. Larrea‐Alcázar, R. Fortunato, B.O. Saidman, J.C. Vilardi.

## DATA ACCESSIBILITY

Upon article acceptance, original data including sampling locations, and microsatellite genotypes will be archived in the public repository Dryad.
